# Words are not just words: how the use of media language in the COVID-19 era affects public health

**DOI:** 10.4178/epih.e2021072

**Published:** 2021-09-23

**Authors:** Georgios P. Georgiou

**Affiliations:** 1 Department of Languages and Literature, University of Nicosia, Nicosia, Cyprus; 2 Department of Foreign Languages, RUDN University, Moscow, Russia

**Keywords:** Epidemiology, COVID-19, Public health, Language

## Abstract

**OBJECTIVES:**

Language can shape the way we perceive the world. In this paper, we investigated how exposure to media texts containing alarming and militaristic language affects peoples’ notions regarding coronavirus disease 2019 (COVID-19) and the consequences of this effect for public health.

**METHODS:**

After reading a passage including either alarming and militaristic or neutral terminology on COVID-19, participants completed a questionnaire in which they answered 4 questions on a 7-point Likert scale. The questions assessed participants’ notions on the end of the pandemic, vaccine effectiveness, and the consequences of COVID-19 for economies and mental health. Ordinal regression models in R were used for the analysis.

**RESULTS:**

Individuals who were exposed to alarming and militaristic language expressed more pessimistic notions regarding COVID-19 than those who were exposed to more neutral language. However, both groups of individuals had similar notions regarding vaccine effectiveness.

**CONCLUSIONS:**

The media should redefine the language they use for the description of the pandemic, considering that the extensive use of alarming and militaristic terminology may have a negative impact on public health.

## INTRODUCTION

Coronavirus disease 2019 (COVID-19) was first reported in December 2019 in Wuhan, China and since then has widely spread throughout the world [1]; shortly before 2022, there were more than 5.3 million deaths and 270 million infections. The COVID-19 pandemic has received intense media coverage that has been substantially different from the coverage of other viral diseases [2,3]. The World Health Organization used the term “infodemic” to describe this massive information bombing, through an analogy with “pandemic.” Although information from the media keeps the public updated about the pandemic, the language used also contributes to the spread of fear among the public [4] since people often read news headlines that characterize COVID-19 as a *deadly disease* and highlight the catastrophic consequences of the pandemic for world economies and humans’ mental health.

Militaristic metaphors are widely used by the media to describe epidemics [5-7]. These metaphors are also prominent in the description of COVID-19, and they usually refer to a *battle* against a deadly virus in which *soldiers* bravely *fight* with every *weapon*; this terminology collectively reminds us of a *war* situation. The use of such metaphors to describe viral diseases is not an unprecedented phenomenon observed only in the COVID-19 era. For example, Basnyat & Lee [8], who investigated the framing of the influenza A (H1N1) pandemic, found that a Singaporean newspaper used several militaristic words. The authors concluded that these words were employed to highlight social responsibility, the risk to the nation due to an imported disease, and alertness over lockdown policies. The language employed in the media can also be alarming. That is, it may use negative descriptions of the virus (e.g., *deadly*), highlight its negative outcomes (number of patients, hospitalizations), or use warnings or negative perlocutionary force markers to frighten or threaten [9].

War metaphors are used when a disease poses a threat to a nation [10]. For instance, war metaphors were not used to a large extent in the United Kingdom media during the severe acute respiratory syndrome (SARS) outbreak since the virus did not threaten the country, but they were extensively used in the Chinese media considering that China was the epicenter of the virus [10]. Although militaristic metaphors can maintain public vigilance and secure the compliance of civilians with the measures during a pandemic, they have some disadvantages. For instance, they encourage control and domination by policymakers, resulting in impingement upon people’s rights [11,12]. Militarization of society violates the values of democracy [13] and is based on the use of authoritarian logic to confront the outcomes of the pandemic [14].

The use of language may affect the way we perceive the world. Kelly & Westerhoff [15] investigated whether the terms “substance abuser” versus “having a substance use disorder” cause different judgments regarding self-regulation, social threat, and treatment versus punishment. In their study, 516 clinicians were asked to read a vignette including 1 term of the 2 terms and completed a questionnaire. The authors found that clinicians who read the term “substance abuser” agreed to a larger extent in that the character was guilty and should be punished. This demonstrates that language can shape the way we encounter individuals or the world in general, creating either positive or negative notions. Aslam et al. [16] examined the sentiments and emotions evoked by news headlines about COVID-19. They used valence shifters on global English headline news from various sources and classified them as positive, negative, and neutral according to the sentiments they evoked. The results showed that the majority of news headlines elicited negative emotions to the readers; specifically, the predominant emotions evoked were fear, trust, anticipation, sadness, and anger. The authors concluded that such sentiments may negatively affect mental health, increasing chronic stress and exacerbating mental disorders, lead to economic disaster, and cultivate stigma and xenophobia.

While numerous studies have emphasized the effects of militaristic language on public freedom and confinement, in this study we provide evidence that alarming and militaristic language may also have an impact on certain notions regarding COVID-19, having short- and long-term negative effects on people and society. Therefore, this study aims to investigate the effect of alarming and militaristic language on public attitudes about COVID-19. Two groups of participants completed a survey that included 1 of 2 different introductory passages: a passage including alarming and militaristic language or a passage including neutral language on COVID-19. They were then asked to indicate the extent to which they agree with 4 statements about COVID-19 and its consequences; this revealed the effect of language use on their notions. The results offer useful insights into public health policy.

## MATERIALS AND METHODS

### Surveys

Two online surveys were created. The instructions asked participants to initially read a passage about COVID-19 and then select the extent to which they agree with 4 statements. The text was at the beginning of the survey and was followed by the statements. The choice of the statements was based on a pilot study that asked 8 individuals to express their concerns and thoughts about COVID-19. The surveyors formulated some predictions about the end of the pandemic and the vaccines and they expressed their concerns on the effect of the disease on the global economy and mental health. These responses were also benchmarked against popular news outlets around the world. On the basis of the pilot study and the general concerns of news outlets, the following statements were formed: (1) *I believe that the pandemic will end soon*, (2) *I believe that vaccines are helpful against COVID*, (3) *I believe that the pandemic will affect economies to a great extent*, and (4) *I believe that the pandemic will affect mental health to a great extent*. Information was also collected about the participants’ gender and educational background.

In each survey, only the passage differed. The passage of the first survey used alarming and militaristic terms to describe the pandemic. For example, it included words such as *deadly, battles, outbreak*, and *threat*, which are characterized by a negative sentimental load [16]. The passage of the second survey included words with a neutral connotation load such as *novel, confronts, onset*, and *challenge*. To cross-validate the appropriateness of the selected opposed words in the 2 surveys, we referred to the database of Warriner et al. [17]. This database presents the mean values of normed valence (the pleasantness of a stimulus) of approximately 14.000 English words as they were rated by speakers (1 [unhappy] to 9 [happy]).

The word *pandemic* was not included in the database, but the similar word *epidemic* evoked unpleasantness to the speakers. We selected *COVID* as its opposed word (this word was also not included in the database) since acronyms are usually used for euphemistic purposes for the description of a disease [18]. Table 1 illustrates the opposed words used in the 2 passages and their mean valence rating. There was a large difference in the mean valence scores of the corresponding words in the two passages. The score of words in Passage 1 ranged from 1.68 to 3.52, while the score of words in Passage 2 ranged from 4.35 to 7.05 (except *illness*). This means that the words of the former passage evoke negative feelings, while the words of the latter passage evoke positive feelings. The surveys were provided in the English language. The passages were as follows:

Passage 1: “A deadly disease officially named COVID-19 was initially reported in Wuhan, China in December 2019. Since then, the world has battled COVID-19. The first vaccine was administered to the public in December 2020, 1 year after the outbreak of the pandemic. Today there are a lot of vaccines against this threat. Scientists worry that the outcomes of the pandemic will be devastating for the economies and humanity, in general, leaving behind millions of unemployed people, and having a dramatic increase of mental diseases.”

Passage 2: “A novel illness officially named COVID-19 was initially reported in Wuhan, China in December 2019. Since then, the world has confronted COVID-19. The first vaccine was administered to the public in December 2020, 1 year after the onset of COVID. Today there are a lot of vaccines against this global challenge. Scientists believe that the outcomes of corona will affect the economies and humanity in general, especially in the domains of employment and mental health.”

### Participants

We surveyed 102 adult participants (n_women_=69; n_men_=33) with different educational backgrounds (n_highschool_=15; n_BA/MA_=70; n_PhD_=17). The questionnaire also solicited information on peoples’ usual reading material or medium (n_broadsheet newspapers_=12; n_social media news_=75; n_news applications_=15). The surveys were randomly administered online through the Facebook platform; specifically, we posted the questionnaire in public health groups from March 20 until April 5, 2021. Most of the participants were residents of European or other Western countries (e.g., United Kingdom, United States, Greece, Cyprus, etc.). Fifty participants completed the questionnaire that included alarming and militaristic terminology (henceforth, militaristic), while another 52 participants completed the questionnaire including relatively neutral language (henceforth, neutral). Participants who believed that there is no pandemic were asked to ignore the questionnaire. Each participant completed only 1 survey.

To control the effects of bias according to factors such as gender, educational background, and reading material/medium, we used chi-square tests to analyze the demographic homogeneity of the participants of the 2 surveys. The results showed no significant differences for any of the variables: gender: χ(1)= 0.56, p=0.45; educational background: χ(2)=4.77, p=0.09; reading material/medium: χ(2)=5.14, p=0.07.

### Statistical analysis

We used ordinal regression models in R [19], which allow probabilistic interpretation of the results. Specifically, we used the MASS package [20] and the polr function. The dependent ordinal variable in each model included a 7-point scale (1, strongly agree; 2, agree; 3, somewhat agree; 4, neutral; 5, somewhat disagree; 6, disagree; and 7, strongly disagree); one for each of the 4 statements. For easier interpretation of the results, we merged the responses into 3 broader response domains: agree, neutral, and disagree. Group (militaristic, neutral) was the categorical factor. We employed the lsmeans package for pairwise comparisons [21].

### Ethics statement

Participants were informed that their participation would be anonymous. They gave their written consent to participate in the questionnaire.

## RESULTS

**Figure 1.**
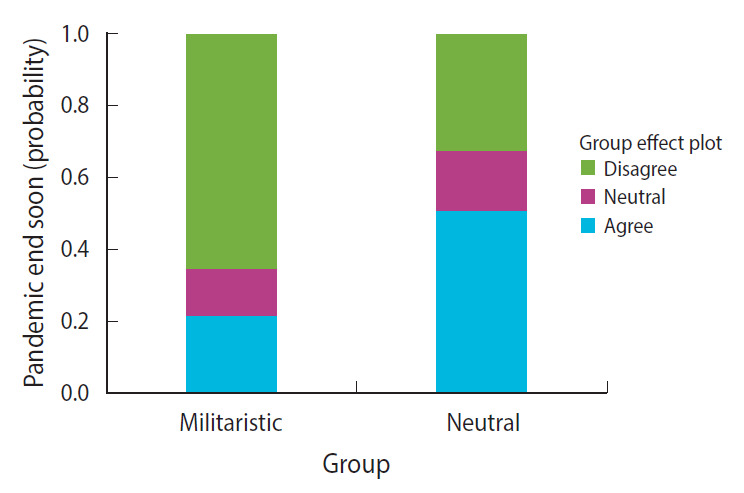
Probabilities of agreement that the pandemic will end soon.

**Figure 2.**
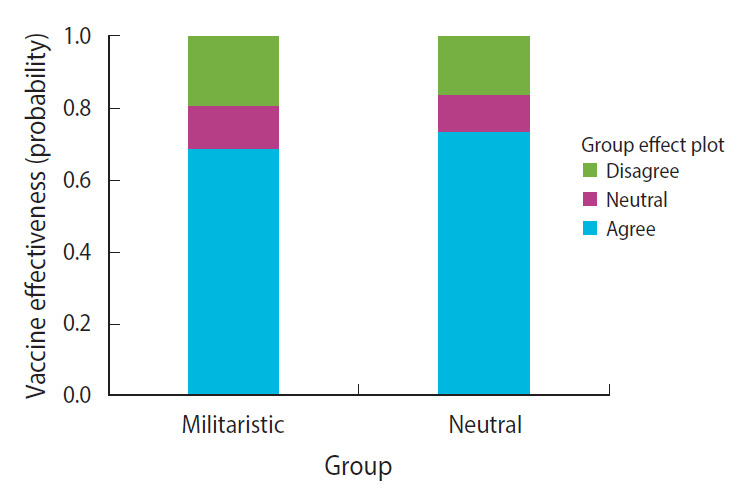
Probabilities of agreement regarding vaccine effectiveness.

**Figure 3.**
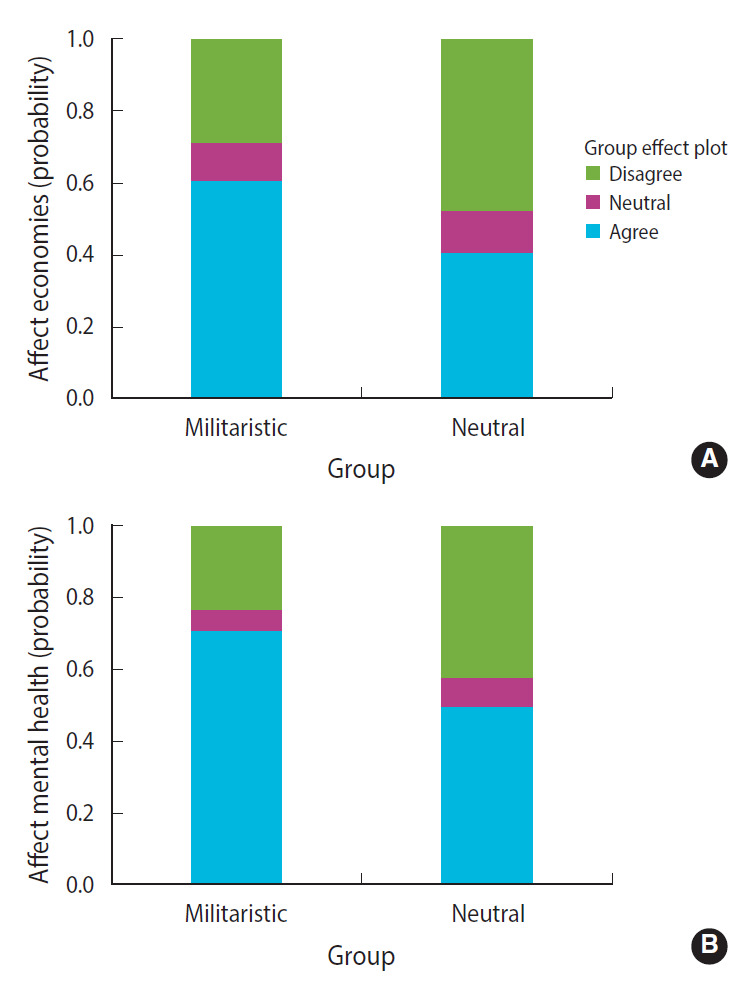
Probabilities of agreement that the pandemic will affect economies (A), mental health (B).

The results showed that the neutral group had a higher likelihood of agreeing that the pandemic will end soon. The probability of agreeing was 0.21 for the militaristic group and 0.51 for the neutral group, and this difference was statistically significant (β, –0.297; standard error [SE], 0.08; p<0.001) (Figure 1). With respect to the effectiveness of vaccines, both groups had a similar likelihood of agreement (Figure 2). It was also observed that the neutral group had a higher likelihood of disagreeing that economies will be affected by COVID-19 to a great extent. The probability of disagreeing was 0.29 for the militaristic group and 0.47 for the neutral group (β, –0.187; SE, 0.089; p=0.035) (Figure 3A). Finally, the neutral group had a higher likelihood of disagreeing that the pandemic will affect mental health to a great extent. The probability of disagreeing was 0.23 for the militaristic group and 0.42 for the neutral group, which was a statistically significant difference (β, –0.192; SE, 0.086; p=0.025) (Figure 3B). The results of the ordinal regression models are shown in Table 2.

## DISCUSSION

The aim of this paper was to investigate the effect of alarming and militaristic language on public attitudes about COVID-19. To this purpose, the participants completed a questionnaire in which they were asked to read a passage including either alarming and militaristic or neutral terminology describing COVID-19 and then answer 4 questions. The responses to the questions were on a Likert scale (1 [strongly agree] to 7 [strongly disagree]) and assessed the participants’ notions on the end of the pandemic, vaccine effectiveness, and the consequences for economies and mental health.

The results demonstrated that individuals who were exposed to written texts with alarming and militaristic language expressed more pessimistic notions regarding COVID-19 in comparison to those who were exposed to more neutral language. These results are consistent with the findings of Aslam et al. [16], who found that news headlines create negative-polarity sentiments in readers. In this study, participants with exposure to frightening language were less likely than those in the other group to agree that the pandemic will end soon and more likely to agree that the pandemic will affect both economies and mental health. However, both groups had similar notions regarding the effectiveness of vaccines, with high percentages of agreement. Perhaps the fact that at the time they completed the questionnaire there was some initial evidence that vaccines could significantly reduce the spread of the virus [22] led participants to express agreement with this item.

The findings manifest how important language can be for public health. The terminology used either verbally or in writing may shape the way individuals encounter real-world situations. Metaphors determine how we understand different phenomena [23] and they also shape our imagination and sentiments [24], consequently affecting our attitudes. It is observed that alarming and militaristic language during a pandemic not only spreads fear, as has been previously shown, but it can also affect the mental health of people by leading them to think more gloomily. This is sensible as they construct images of war in their mind. Therefore, the mental health of people is burdened through exposure to fearful language. Maintaining good mental health during a pandemic is important considering that there have been reports that a significant proportion of the population during the COVID-19 pandemic was categorized as being at high risk for committing suicide [25]. Another important aspect is the potential economic disaster as a consequence of pessimistic notions, which may result in reduced demand for products and investments with a subsequent increase in unemployment (for extended discussion, see [16]).

There have been calls to replace military metaphors with more attractive ones [10], as such metaphors can increase political and authoritarian power or enhance social sigma. We will add another point to consider: military metaphors and alarming language can negatively affect public health and especially mental health. Therefore, the media should be very careful with the language they use, decreasing the inclusion of militaristic metaphors and replacing them with more neutral terms. Although the sample of the study was representative, a higher number of participants would have yielded more precise results on how language use affects notions about COVID-19. Furthermore, although we tried to control as many factors as possible, some other factors, such as the surveyors’ political affiliations and cultural characteristics may have affected the results; these issues could be considered in a future study.

## Figures and Tables

**Table 1.** Words used in the 2 passages and their mean valence scores in the parenthesis (1 [unhappy]-9 [happy])

Passage 1		Passage 2	
Words	Score	Words	Score
Deadly	1.90	Novel	5.74
Disease	1.68	Illness	1.95
Battles	3.52	Confronts	4.59
Outbreak	2.47	Onset	4.35
Pandemic	-	COVID	-
(Epidemic)	(2.05)		
Threat	2.63	Challenge	5.95
Worry	2.10	Believe	7.05
(Be) devastating	2.09	Affect	5.65

**Table 2.** Results of the ordinal regression models

Groups	Value Std.	Error	t-value	p-value
Pandemic will end soon				
Neutral	-1.355	0.401	-3.376	0.001
Agree/neutral	-1.328	0.319	-4.160	<0.001
Neutral/disagree	-0.642	0.296	-2.169	0.030
Vaccines are effective				
Neutral	0.233	-0.436	-0.534	0.593
Agree/neutral	0.784	0.299	2.618	0.009
Neutral/disagree	1.406	0.328	4.287	<0.001
Pandemic will affect economies				
Neutral	0.807	0.393	2.055	0.040
Agree/neutral	0.454	0.290	1.568	0.117
Neutral/disagree	0.922	0.301	3.064	0.002
Pandemic will affect mental health				
Neutral	0.892	0.414	2.154	0.031
Agree/neutral	0.889	0.316	2.818	0.005
Neutral/disagree	1.206	0.326	3.696	<0.001
